# Case Studies: Use of Salicylic Acid (Avosil) and Hydrogel (Avogel) in Limiting Scar Formation

**Published:** 2005-03-28

**Authors:** John R. Danielson, Robert J. Walter

**Affiliations:** a St. Mary's Hospital, Hobart, IN; b John Stroger Hospital of Cook County, Department of Trauma, Chicago, IL

## Abstract

**Objective:** The purpose of this report is to present the results of a preliminary treatment regimen for hypertrophic scars combining topical 2% salicylic acid cream (Avosil) with an overlay of hydrogel dressing (Avogel). **Methods:** The study group consisted of 3 patients with symptomatic hypertrophic scars: 2 presternal and 1 on the inner thigh. Scars were divided into 3 equal-size areas: (1) untreated control, (2) hydrogel alone, and (3) 2% salicylic acid with hydrogel cover. Treatments were applied every 8 to 12 hours and a Velcro appliance was employed to cover the area during treatment. The total length of treatment was 60 days. **Results:** At the end of the 60-day treatment protocol, the area treated with 2% salicylic acid and hydrogel was asymptomatic. In contrast, the hydrogel-treated and untreated control areas remained erythematous and symptomatic for burning pain and pruritis. **Conclusion:** This small study suggests the efficacy of combined salicylic acid and hydrogel therapy in the treatment of hypertrophic scars. More extensive studies of scar treatment with salicylic acid and hydrogel are needed. These studies must be larger in scope to carefully document the spectrum of patient responses and should include methods for evaluating alterations in the levels of different inflammatory mediators.

Thirty years ago, Larson et al observed that elastic pressure wrap dressings were effective in reducing scar hypertrophy in healing burn scars.^1^ Since that report, 20 to 24 mm Hg pressure garments have become the mainstay of scar prevention. The mechanism of action of pressure dressings is uncertain, particularly in view of the fact that they remain effective following several weeks of daily use despite a loss of elasticity and a diminution of pressure exertion. Biochemical assays of tissue healing beneath pressure garments show moderate decreases in wound metabolism and increased collagenase activity.^2^ However, pressure garments are poorly tolerated because of the general discomfort and restriction of movement that accompanies their use.

In the past decade, another topical device, silicone gel sheeting, has been found to be useful clinically in controlling scar formation.^3^–^5^ As with elastic garments, the mechanism of action has not been convincingly elucidated but postulated mechanisms include induction of scar hypoxia, increased hydration of the epidermis covering the scar,^6^ and increased scar temperature.^7^,^8^ The effect does not depend on the composition of the gel sheeting used, and it is clear that release of silicone into the scar is not the mode of action. Recently, several reports have shown that hydrogel sheeting is just as effective as silicone but has fewer adverse side effects.^9^,^10^ Hydrogel sheeting has been approved by the Food and Drug Administration and is considered to be substantially equivalent to silicone for treatment of hypertrophic scars. Thus, the effect of the topical gel sheeting seems independent of its composition. Hydrogels have the added advantages of being useful as drug-delivery vehicles and of having higher heat capacities for buffering scar temperature.^8^

Inflammation is critically important for healing and scarring. Dang et al^11^ and O'Kane^12^ recently reviewed the biology of scar formation. Both reviews underscore the importance of inflammation as the essential epigenetic stimulator of scarring. Therefore, we performed a pilot investigation of topical salicylic acid (2%) treatment in conjunction with hydrogel sheeting to determine whether there is an added beneficial effect from using a topical nonsteroidal anti-inflammatory drug in scar management. Here, we report our experience with the first patients.

## METHODS

The first 2 cases were part of an ongoing pilot study at St. Mary's Hospital designed to document the effect of topically applied salicylic acid on symptomatic hypertrophic scars. Both patients had tender and pruritic hypertrophic scars following median sternotomy. For study inclusion, the length of the scar needed to be sufficient to accommodate a control area and 2 experimental areas (each of equal size). The control area was left untreated while one experimental area was covered with Avogel hydrogel (Avocet Polymer Technologies, Inc, Chicago, Ill) and a second area of equal length was treated with 2% salicylic acid in Eucerin (Beiersdor, Wilton, Conn) and covered with Avogel hydrogel. The study regimen called for treatments to be performed for 8 to 12 hours per day employing a Velcro appliance specially constructed to cover the scar during treatment and to prevent salicylic acid from spreading into the control areas. Exclusion criteria included the use of anti-inflammatory agents during the previous 2 weeks or known allergies to anti-inflammatory agents. The scars were assessed at 2-week intervals for 60 days. Direct measurements, histories, and digital photographs were obtained. An end stage was achieved when the experimental region of the scar was flat and asymptomatic and when erythema had receded.

The third patient was treated in a similar fashion by a Chicago area plastic surgeon for an 11-month-old hypertrophic scar from a burn injury on the inner thigh. This case is included to demonstrate that similar effects have been observed independently. The protocol used to treat this patient is described below.

### Ethics

The Institutional Review Board of St. Mary's Hospital approved this study, and all participants gave informed consent. The procedures followed were in accordance with the ethical standards as set forth by the institution and with the Helsinki Declaration of 1975, as revised in 1983.

## RESULTS

All patients experienced significant symptomatic relief from tenderness and pain within days in the area of the scar treated with 2% salicylic acid and hydrogel. Some relief in symptoms was recorded in the area treated with hydrogel alone, but no symptomatic relief was experienced in the untreated area. Noticeable decreases in scar width and height were noted in all patients over the 60-day study period in the area treated with salicylic acid and hydrogel. Comparatively small changes in scar dimensions were noted with hydrogel alone, and no change was noted in the control region. Toward the end of the study period, scar erythema was greatly decreased in the area treated with hydrogel and salicylic acid in all patients.

## CASE REPORTS

### Case #1

**Figure 1:**
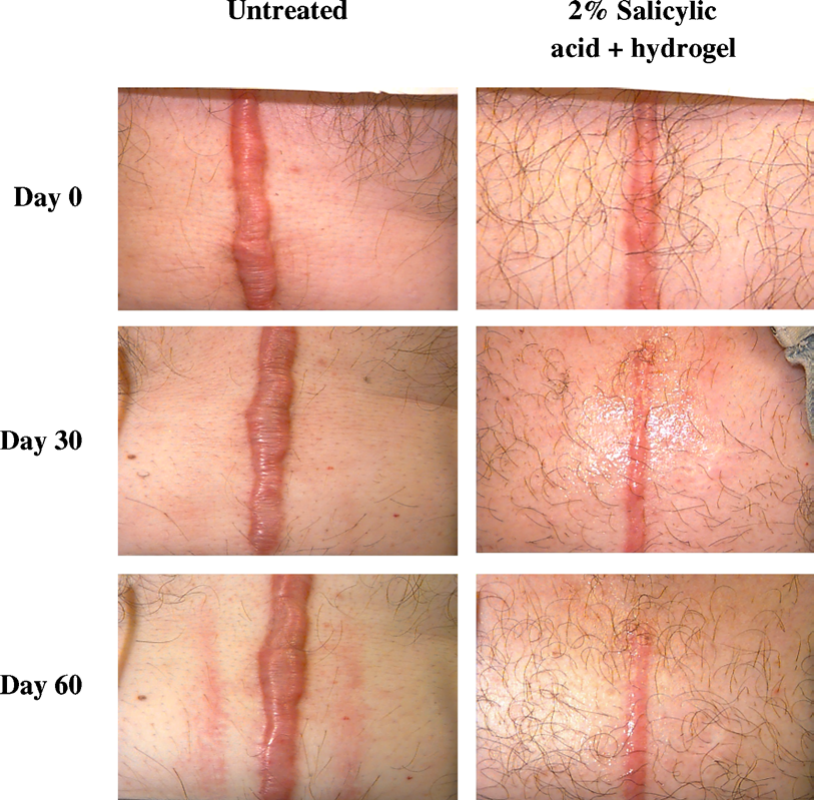
This patient is a 49-year-old man who underwent open-heart surgery 6 months prior to presentation at our clinic. At the end of the 60-day treatment protocol, the area treated with 2% salicylic acid and hydrogel was asymptomatic and showed reduced erythema, whereas the hydrogel-treated and untreated control areas remained erythematous and symptomatic for burning pain and pruritis.

This patient is a 49-year-old man (Fig 1) who underwent open-heart surgery 6 months prior to presentation at our clinic. The patient's scar was 22 cm long, varied from 5 to 8 mm in width, and was elevated by 2 to 3 mm throughout. He complained of tenderness to touch and pain. After starting on the treatment protocol, all symptoms abated in the area treated with 2% salicylic acid and hydrogel within 2 weeks. During subsequent weeks, the scar height of the experimentally treated area decreased dramatically such that the scar became completely flattened to the level of the adjacent tissue. The control area remained unchanged, and the experimental area treated with hydrogel alone did not resolve as markedly as the area treated with 2% salicylic acid and hydrogel together. Furthermore, the degree of erythema decreased in the area treated with 2% salicylic acid and hydrogel, whereas the scar color remained unchanged in the area treated with hydrogel alone. At the end of the 60-day treatment protocol, the area treated with 2% salicylic acid and hydrogel was asymptomatic. In contrast, the hydrogel-treated and untreated control areas remained erythematous and symptomatic for burning pain and pruritis.

### Case #2

**Figure 2:**
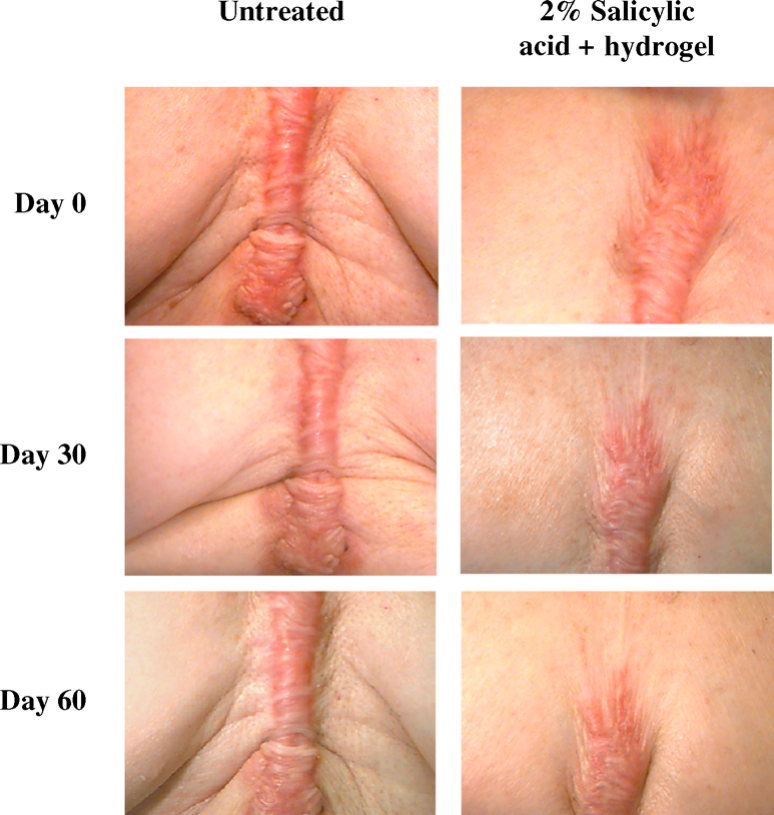
This patient is a 65-year-old woman who underwent open-heart surgery 2½ years prior to presentation at our clinic. After 60 days of treatment, her symptoms in the area treated with 2% salicylic acid and hydrogel had completely abated. The hydrogel-treated and untreated control areas remained symptomatic.

This patient is a 65-year-old woman (Fig 2) who underwent open-heart surgery 2½ years prior to presentation at our clinic. Before the study, she had applied vitamin E topically to the scar for a short period of time without effect. She complained of pain and tenderness, and the scar was very elevated and erythematous. The patient's scar was 14 cm long, varied from 4 to 6 mm in width, and was elevated by 3 to 4 mm. Symptomatic relief from pruritis and tenderness occurred within 48 hours of applying salicylic acid and hydrogel. One week after beginning the study regimen, she noted a marked decrease in symptoms and decreased erythema in this area. Regression in the size of the scar was less impressive, but symptomatic relief was significant. After 60 days of treatment, her symptoms in the area treated with 2% salicylic acid and hydrogel had completely abated. The hydrogel-treated and untreated control areas remained symptomatic.

### Case #3

**Figure 3:**
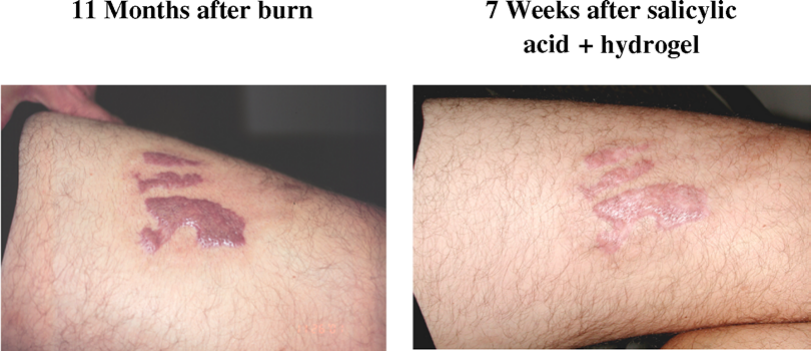
This patient is a 42-year-old man seen 11 months after receiving a burn on the thigh. A hypertrophic scar was evident (left panel) and the patient reported intense pruritis. In addition, the scar was very elevated and erythematous. On 7-week follow-up after treatment with salicylic acid and hydrogel (right panel), the scar showed a notable decrease in redness and thickness and the patient reported diminished pruritus.

This patient is a 42-year-old firefighter who suffered a flame burn injury to the skin on the left inner thigh 11 months before presentation. He was treated as an outpatient at one of the burn centers in Chicago where he received topical anti-microbials and dressing changes. The wound healed by epithelialization and hypertrophic scarring. The patient was referred to the University of Chicago Scar Clinic because the burn scar had become quite hypertrophic and was unresponsive to compression stocking therapy. A photograph of the scar taken at that time is shown in Figure 3 (left). Using a visual analog scale of scar pruritus, the patient scored itch intensity at 6/10 (where 10 = unbearable itching) and itch frequency at 6/10 (where 10 = itching at all times). With time, the scar continued to progressively increase in thickness.

Therapy was initiated with Avogel hydrogel with 3% salicylic acid in a cream base applied to the scar each night and left for 8 to 10 hours. The use of compression garment therapy was discontinued. One month later, treatment regimen was adjusted such that 2% acetylsalicylic acid in Eucerin ointment (Beiersdor, Wilton, Conn) with hydrogel was used to achieve better symptomatic relief. On 7-week follow-up (Fig 3, right), the patient showed a decrease in scar redness and thickness and a 50% reduction in the intensity of pruritus. Scar symptoms were exacerbated by work activity, in which heavy clothing abraded the scar, but on 16-week follow-up, the scar manifested considerable involution and the intensity of itching was scored as 3/10. The patient continues to be treated with salicylic acid (left overnight for 8 hours).

## DISCUSSION

Limiting inflammation is paramount in the control of scar growth and scar-associated symptoms. The inflammatory response can be regulated at several different physiological levels. Three common ways that inflammation can be controlled are by inhibiting cytokine production via cyclooxygenase (COX) regulation, inhibiting histamine binding to its receptors, and inhibiting the NF-κB signal that upregulates inflammation. The most widely used anti-inflammatory agents belong to the broad category of nonsteroidal anti-inflammatory drugs. They inhibit either prostaglandin production or NF-κB generation or both. It is noteworthy that steroids also exhibit anti-inflammatory effects by inhibiting inflammatory response gene promoters NF-κB, AP-1 (activator protein-1), and NF-AT (nuclear factor of activated T lymphocytes).^13^

The most common mode of prostaglandin synthesis blockade is inhibition of cell membrane–bound COX. There are 2 isoforms of COX expressed in human cells, COX-1 and COX-2.^14^ COX-1 is expressed constitutively throughout the body, especially in stomach and kidneys. On the other hand, COX-2 is expressed constitutively in the brain and kidneys.^15^ COX-2 is highly inducible at sites of inflammation, playing an important role in fibrosis. Many adverse side effects of COX inhibition are minimized by use of specific COX-2 inhibitors, making them agents of choice for prolonged usage. The therapeutic value of these agents in many rheumatologic diseases is well established. However, their potential value in management of hypertrophic scarring has been a more recent consideration.^16^ NF-κB is a rapid response transcription factor that is involved in stress responses and is central to the inflammatory reaction.^17^ NF-κB is involved in the upregulation of both cell membrane receptors to inflammatory peptides and the production of cytokines, chemokines, and growth factors. NF-κB activation can be inhibited by several different agents, including cyclosporine, tacrolimus, antioxidants, and salicylates (including aspirin). Salicylic acid inhibits NF-κB expression by blocking the dissociation of IκB (the inactivator of NF-κB) from NF-κB in the cytoplasm and thus decreases the amount of inflammation that occurs.^17^ At concentrations of 2% to 5%, salicylates are commonly used to control skin inflammation and are routinely used in over-the-counter acne remedies.

Antihistamines are commonly used only to control the symptoms of scar pruritus. However, they have other important effects that may function to reduce scarring. Antihistamines, particularly H_1_ blockers, inhibit the inflammatory response, resulting in reduced scar formation and reduced discomfort. Patients scratch the inflamed scar less frequently, which probably reduces scar growth rate. Finally, antihistamines are well known to inhibit collagen synthesis.^18^ Benadryl and Atarax are the most commonly used antihistamines for scar management. In the past few years, we have preferred the use of long-acting, nondrowsy formulations such as loratadine (Claritin; Schering, Kenilworth, NJ) or fexofenadine (Allegra; Aventis, Kansas City, Mo), which have the advantages of sustained action and fewer central nervous system side effects. In recent studies, topically applied aspirin has been found to decrease histamine-induced wheal and flare reactions.^19^ However, topically applied salicylic compounds did not diminish serotonin-induced scratching behavior in rats.^20^

From the above, it is clear that more extensive studies of scar treatment with salicylic acid and hydrogel are needed. These studies must be larger in scope to carefully document the spectrum of patient responses and should include methods for evaluating alterations in the levels of different inflammatory mediators.
